# Mammography and ultrasound effective features in differentiating basal-like and normal-like subtypes of triple negative breast cancer

**DOI:** 10.18632/oncotarget.19053

**Published:** 2017-07-06

**Authors:** Zeng Zeng, Chun Jie Hou, Qiao Hong Hu, Ying Liu, Ceng Wang, Ran Wei, Xiao Ming Fan

**Affiliations:** ^1^ Department of Ultrasound, Zhejiang Provincial People’s Hospital, Hangzhou, Zhejiang, China

**Keywords:** triple negative breast cancer, ultrasound, mammography

## Abstract

The aim of our study was to find effective features of mammography and ultrasound in differentiating Basal-like breast cancer (BBC) and Normal-like breast cancer (NBC), two subtypes of triple negative breast cancer (TNBC). From January 2014 to March 2017, we retrospectively reviewed 91 patients who were pathologically confirmed as TNBC. According to immunohistochemical cytokeratin 5/6 (CK5/6) and Epidermal Growth Factor Receptor (EGFR), TNBCs were classified into BBCs group and NBCs group. Both CK5/6 and EGFR were negative defined to be NBC, whereas if any of CK5/6 or EGFR was positive then defined as BBC. BBCs group concluded 65 (71.4%) cases and NBCs group concluded26 (28.6%) cases. Ultrasound images and mammograms were reevaluated by breast imaging experts according to the breast imaging reporting and data system (BI-RADS) 4th edition. On mammography, masses margins had significant differences between BBCs group and NBCs group (*P* = 0.024). Most BBCs margins exhibited microlobulated (30/64, 46.9%) or spiculated (25/64, 39.0%), whereas most NBCs margins exhibited microlobulated (17/23, 73.9%). On ultrasound, BBCs were more frequently to present as larger than 20mm lesions (52/65, 80.0%) and more likely to have angular or spiculated margins (35/65, 53.8%), additionally, compared with NBCs, BBCs were less likely to have calcification (1/65, 1.5%). Other mammography and ultrasound features showed no significant differences between the two groups. In conclusion, we have found some effective features of mammography and ultrasound that could be helpful in differentiating BBC and NBC, which will provide some useful references for clinical diagnosis and treatment.

## INTRODUCTION

Triple-negative breast cancer (TNBC) is defined as a distinct subtype of breast cancer which lacks expression of estrogen receptor (ER), progesterone receptor (PR), and human epidermal growth factor receptor 2 (HER2). TNBC always demonstrates poor prognosis with recurrence in a short survival time because of its aggressive tumor biology character [1]. Surgery represents the optimal modality for local control of TNBC. However, though adding chemotherapy or radiotherapy after surgery, recurrences or metastases always occur [2]. Nowadays chemotherapy is the only modality of systemic therapy for TNBC patients. According to the gold standard microarray expression profiling analysis, TNBC can be divided into two subtypes, the one is basal-like breast cancer (BBC) and the other is normal-like breast cancer (NBC) [3]. BBCs account for 15% of all breast cancer and 85% of TNBCs [4, 5]. Neoplastic cells express genes consistently in BBCs, such as immunohistochemical cytokeratin 5/6 (CK5/6) and epidermal growth factor receptor (EGFR) [6–9]. NBCs do not express the gene profile of BBCs and the cells in NBCs are similar to normal mammary stromal cells [4, 5]. Immunohistochemical imaging features of two subtypes are different. BBCs are characterized by high histological grade, high mitotic index, central necrotic zones, pushing borders and conspicuous lymphocytic infiltrate [10, 11]. Moreover, metaplastic elements and medullary/atypical medullary features are significantly more prevalent in BBCs [12]. BBC is associated with a high malignancy potential and poor overall prognosis compared with NBC. Brain and lungs metastasis always occur at an early stage [13–16]. NBC has a slightly better prognosis and not respond to neoadjuvant chemotherapy like BBC do [17–21]. So early detection and classify subtypes are of great significance in clinical.

Previous studies have analyzed mammography and ultrasound findings of TNBC, a lobulated mass, with less attenuating posterior echoes, some vascularity, and low elasticity always indicated TNBC. However, there haven’t been any further study of the TNBC subtypes in image characteristics. Thus, the purpose of our study was to find the effective features of mammography and ultrasound in differentiating the TNBC subtypes of BBC and NBC.

## MATERIALS AND METHODS

### Patients

This study was approved by the ethical and scientific review board of the Zhejiang Provincial People’s Hospital. From January 2014 to March 2017, we retrospectively reviewed 91 patients who were pathologically confirmed as triple negative breast cancer after surgery. Written informed consents were omitted from our scientific review board because our study was retrospective and all the patients’ information was anonymized. Patients (1) examinations of breast mammography and ultrasound were performed before any treatment or surgeries; (2) immunohistochemical character of ER, PR, HER-2, CK5/6 and EGFR could be obtained; (3) had single and unilateral breast lesions were included in the study. Eventually, 91 breast lesions in 91 patients (0 male and 91 female) met the inclusion criteria.

### Mammography

Standard two-view mammography consisted of a lateral oblique and a craniocaudal view of each breast was performed using GE Senographe 2000D system (GE Healthcare, Milwaukee, Wis). Two breast radiologists each with 3 years of experience in diagnosis breast cancer through mammograms retrospectively reviewed all the patients’ mammograms in 1 month, if any discrepant results occurred, they would discuss and then reach an agreement. According to the Breast imaging reporting and data system (BI-RADS) 4th edition, masses were described as masses only, calcifications only, masses with calcifications, focal asymmetries and architectural distortion; breast density were described as predominantly fatty, scattered fibroglandular, heterogeneously dense and dense [22]. We recorded masses borders as microlobulated, obscured and spiculated.

### Ultrasound

Ultrasound was performed using 5–12 MHz transducers with an HDI 5000 or IU-22 (Philips Medical Systems, Best, the Netherlands) ultrasound unit. Two breast radiologists (each with at least 10 years of clinical experience) read the ultrasound images retrospectively and independently according to the Breast imaging reporting and data system (BI-RADS) 4th edition [22]. If there were any disagreements, then a consensus interpretation must be reached. The size (< 20 mm or < 20 mm), shape (oval, lobulated, polygonal or irregular), boundary (circumscribed, indistinct, angular or spiculated), echo pattern (hypoechoic, isoechoic or hyperechoic), posterior echo (accentuating, no change, attenuating), calcification (yes, no) and color Doppler (avascular, spotty signals, hypovascular, hypervascular) of lesions were recorded. We also recorded the growth orientation and blood flow signals of lesions.

### Pathological findings

Pathological findings were assessed by two experienced pathologists (each 5 and 10 years in pathology of breast) independently. Tissue preserved by buffered formalin and embedded in paraffin blocks. Estrogen receptor (ER), progesterone receptor (PR), human epidermal growth factor receptor 2 (HER2) cytokeratin 5/6 (CK5/6) and epidermal growth factor receptor (EGFR) were evaluated. ER and PR positive expression more than 10% considered to be positive. HER2 status was graded as 0, 1+, 2+ and 3+, 3+ considered as positive. 2+ was checked by fluorescence *in situ* hybridization (FISH) for its positivity and HER2 gene amplification on FISH was considered to be positive. Membrane staining was assessed for EGFR according to DAKO criteria. Any intensity of EGFR in more than 1% of cells was considered to be a positive basal marker, and the detection of CK5/6 cytoplasmic expression in either tumour or surrounding tissues was considered to be CK5/6 positive. In TNBCs, we considered both CK5/6 and EGFR negative to be NBC, whereas if any of CK5/6 or EGFR was positive, we considered them as BBC which was proposed by Nielsen et al. [23]. According to the Scarff-Bloom-Richardson System invasive cancer was graded as grade 1 (well differentiated), grade 2 (moderately differentiated) or grade 3 (poorly differentiated) [24].

### Statistical analysis

We used SPSS software (SPSS for Windows 22.0, SPSS, Chicago, IL) to do some statistical analysis. We used chi-square test for qualitative data and Student *t* test for quantitative data. *P* value less than 0.05 indicated significant difference.

## RESULTS

Ultimately 91 patients whose pathological type were all invasive ductal carcinomas (IDC). Table 1 provides basic information of these patients. BBCs group and NBCs group had no significant differences in age, family history of breast cancer, lymph node metastasis, pathologic type and their histological grade for IDC. However, the two groups had differences in tumour sizes (*P* < 0.001). The mean size of BBCs was larger than NBCs.

**Table 1:** Clinicopathologic findings of 91 patients with TNBC according to CK5/6 and EGFR status

Finding	Basal-like TNBC (CK5/6+ or EGFR+ or both+) *N* = 65	Normal-like TNBC (CK5/6- and EGFR-) *N* = 26	*P* value
Age (y)			0.226
Mean	54.1 ± 8.3	51.8 ± 7.7	
Family history of breast cancer			0.787
Yes	38	16	
No	27	10	
Tumour Size (cm)			< 0.001
Mean	4.2 ± 1.1	1.8 ± 0.9	
Range	2.9−6.0	1.0−3.5	
Histological grade for IDC			
1 or 2	21	11	0.367
3	44	15	
Lymph node metastasis			0.504
Yes	35	16	
No	30	10	

Table 2 shows the mammography findings of TNBCs in our study. BBCs always presented as only masses (47/64, 73.4%), however less associated with calcifications (2/64, 3.1%), masses with calcifications (4/64, 6.3%), focal asymmetries (6/64, 9.4%) or architectural distortion (5/64, 7.8%). Similarly NBCs mostly noted as only masses (16/23, 69.6%) and calcifications only couldn’t be found at any NBCs mammography. Most BBCs margins exhibited microlobulated (30/64, 46.9%), or spiculated (25/64, 39.0%), whereas others showed obscured (9/64, 14.1%). Most NBCs margins exhibited microlobulated (17/23, 73.9%), small part showed obscured (4/23, 17.4%) or spiculated (2/23, 8.7%). Masses margins had differences between two groups and the *P* value was 0.024, however no significant differences in breast density. 4 cases were missed diagnosis by mammography, the one is BBC and the other 3 were NBCs.

**Table 2:** Mammographic findings of 87 patients with TNBC according to CK5/6 and EGFR status (4 cases were missed diagnosis: 1 BBC and 3 NBCs)

Finding	Basal-like TNBC (CK5/6+ or EGFR+ or both+) *N* = 64	Normal-like TNBC (CK5/6- and EGFR-) *N* = 23	*P* value
Masses			0.768
Masses only	47	16	
Calcifications only	2	0	
Masses with calcifications	4	1	
Focal asymmetries	6	4	
Architectural distortion	5	2	
Breast density			0.225
Predominantly fatty	1	0	
Scattered fibroglandular	34	7	
Heterogeneously dense	28	15	
Dense	1	1	
Borders			0.024*
Microlobulated	30	17	
Obscured	9	4	
Spiculated	25	2	

The results of ultrasound were shown in Table 3. We selected characters such as masses sizes, shapes, boundaries, posterior feature, echo patterns, calcifications, growth orientations, Color Doppler and blood flow signals. The masses sizes, boundaries and their calcifications had great significant differences between BBCs and NBCs. Breast masses larger than 20mm were more likely to be seen in BBCs (52/65, 80.0%). Among all the BBCs, masses with angular or spiculated margins were most frequently seen (35/65, 53.8%), indistinct margins (24/65, 36.9%) were commonly observed, but circumscribed margins were rare (6/65, 9.2%). However, masses with circumscribed margins were always seen in NBCs (16/26, 61.5%) and less likely to be seen as indistinct (8/26, 30.8%) or angular/spiculated margins (2/26, 7.7%). We could find only one case of calcification in BBCs whereas 15 cases of calcifications in NBCs.

**Table 3:** Ultrasound findings of 91 patients with TNBC according to CK5/6 and EGFR status

Finding	Basal-like TNBC (CK5/6+ or EGFR+ or both+) *N* = 65	Normal-like TNBC (CK5/6- and EGFR-) *N* = 26	*P* value
Size			< 0.001*
< 20 mm	13	16	
> 20 mm	52	10	
Shape			0.601
Oval	2	3	
Lobulated	15	21	
Polygonal	18	1	
Irregular	30	1	
Boundary			< 0.001*
Circumscribed	6	16	
Indistinct	24	8	
Angular or Spiculated	35	2	
Echo pattern			0.442
Hypoechoic	44	14	
Isoechoic	15	8	
Hyperechoic	6	4	
Posterior echo			0.646
Accentuating	6	1	
No change	19	9	
Attenuating	40	16	
Calcification			< 0.001*
Yes	1	15	
No	64	11	
Color Doppler			0.966
Avascular	18	7	
Spotty signals	10	5	
Hypovascular	17	7	
Hypervascular	20	7	
Growth orientation			0.699
Aspect ratio > 1	15	7	
Aspect ratio < 1	50	19	
Blood flow signals			0.466
Adler 0–1	32	15	
Adler 2–3	33	11	

**Figure 1:**
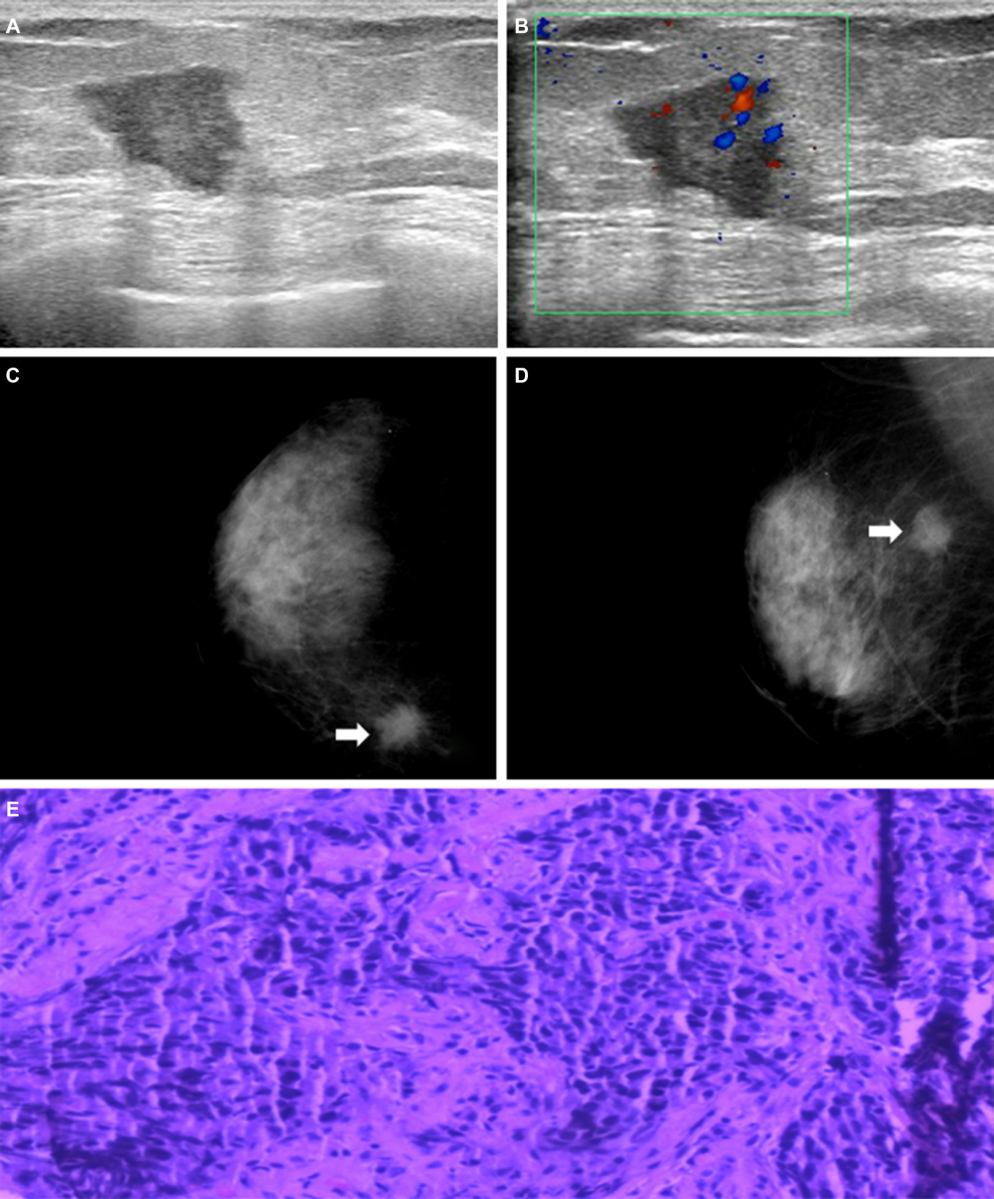
(**A**) and (**B**) Mammogram revealed a high density mass in the inner upper quadrant of the right breast (arrows) and its border was spiculated. (**C**) and (**D**) Ultrasound revealed an irregular shape mass with circumscribed margin in low echoic area. The mass size was 3.2 cm × 2.6 cm. Non-accentuating posterior echoes, non-calcification either. Spotty signals could be seen via Color Doppler. (**E**) Histopathological image showed it as invasive ductal carcinoma, original magnification, 200×; ER-, PR-, HER2 1+, CK5/6- and EGFR- showed it as NBC.

**Figure 2:**
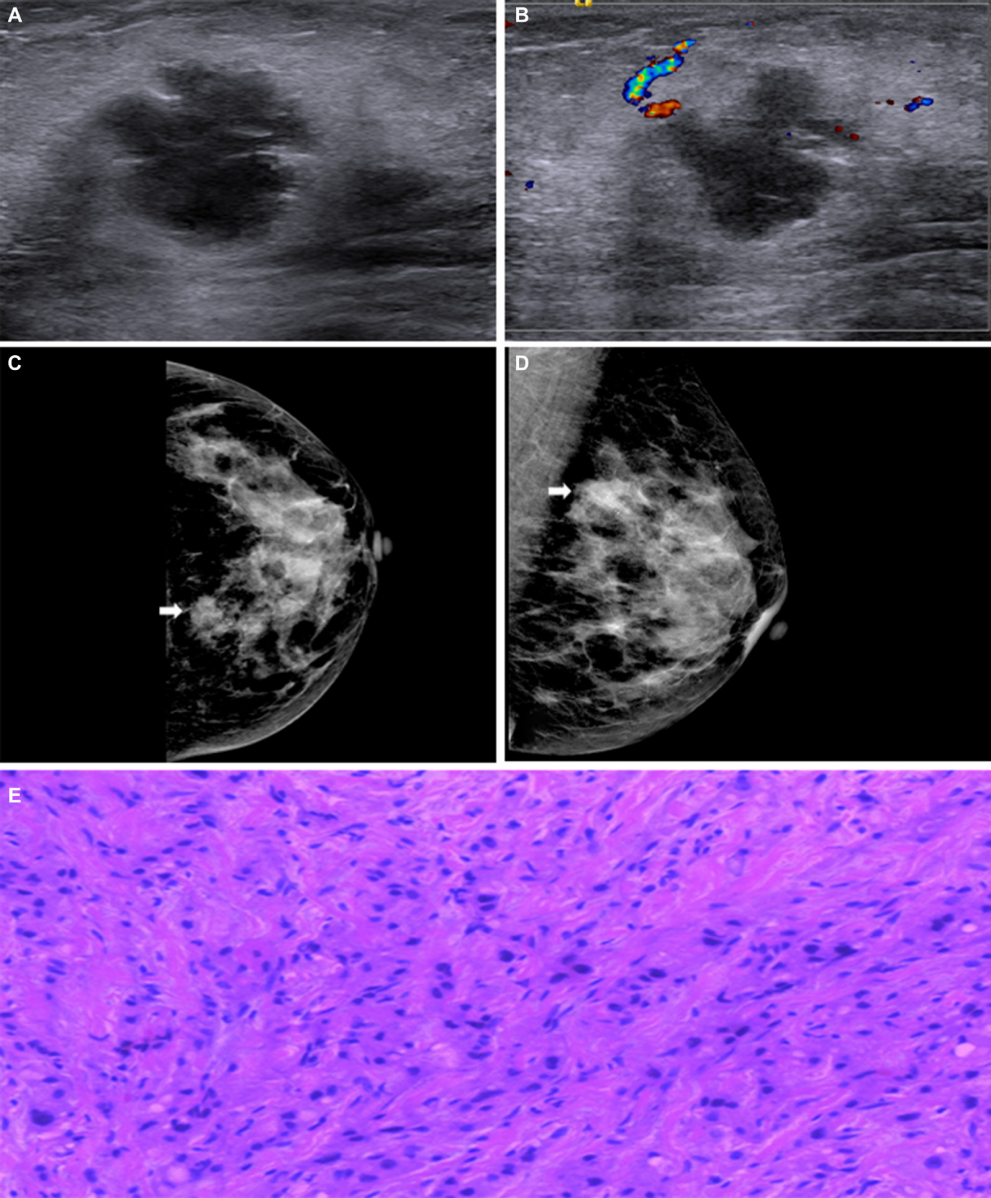
(**A**) and (**B**) Mammography displayed an isodensity mass with circumscribed border in the inner upper quadrant of the left breast (arrows). (**C**) and (**D**) Ultrasound revealed a mass with lobulated margin in hypoechoic. The mass size was 4.0 cm × 2.3 cm. Non-accentuating posterior echoes, non-calcification either. No obvious blood signals in the mass. (**E**) Histopathological image showed it as invasive ductal carcinoma, original magnification, 200×; ER-, PR-, HER2 1+, CK5/6+ and EGFR+ showed it as BBC.

Representative cases are shown in Figure 1 and Figure 2. On the mammogram of the first case, there was a high density mass in the inner upper quadrant of the right breast and its border was spiculated. On ultrasound images we could find an irregular shape mass with circumscribed margin in a low echoic area. The mass size was 3.2 cm × 2.6 cm. The pathological findings confirmed it as invasive ductal carcinoma in nuclear grade 2 with lymphovascular invasion. Immunohistochemical findings were ER negative, PR negative, HER-2 (1+) negative. Because of its CK5/6 and EGFR were both negative, we defined it as NBC. Figure 2 showed an isodensity mass with circumscribed border in the inner upper quadrant of the left Breast. On the ultrasound, there was a mass with lobulated margin in hypoechoic. The mass size was 4.0 cm × 2.3 cm. Its posterior echo wasn’t attenuating and we couldn’t see any calcifications either. Spotty signals could be seen via Color Doppler. The pathological result confirmed invasive ductal carcinoma with a nuclear grade 3. Immunohistochemical findings were ER negative, PR negative, HER-2 (1+) negative. Because of CK5/6 positive, we considered it as BBC.

## DISCUSSION

TNBCs always express aggressive histologic features, high rates of recurrence, distant metastases and survive shorter time compared with other breast cancer subtypes [25]. In our study the median age of BBCs and NBC was 54.1 ± 8.3 and 51.8 ± 7.7 separately, it is similar to the median age of 51 which was conducted by Li et al. in Chinese women [26]. But lower than Turkish or Caucasian patients. The reason may be the diversity of race, menopausal status or weight differences [27, 28]. According to our research, we found some features of all the TNBCs in mammography and ultrasonography. On mammography, TNBCs often seen as an oval mass but less associated with calcifications, this character was similar to the finding of Ko et al., they suggested that triple-negative breast cancers change rapidly into an invasive stage and without a precancerous stage [29]. So calcifications were not always occur. In the ultrasound findings, most of the TNBCs were always seen as an extremely hypoechoic oval mass lesions, lobulated margins and enhancement of posterior echoes. Pathological researchers reported that necrosis like internal fluid component might cause the enhancement of posterior echoes.

Although some researchers have claimed the BBCs compose almost all of triple-negative breast cancers, basal-like breast cancer is not a synonym for Triple-negative breast cancers [30]. BBCs form 56–85% of triple-negative breast cancers [3, 11]. Basal layer of breast epithelium expresses certain gene clusters in basal-like breast cancers. So BBCs always involved in cellular proliferation, suppression of apoptosis, cell migration and cell invasion. Nielsen et al. [31], defined basal-like breast cancers as those expressing both CK5/6 and EGFR. Nielsen’s panel has 100% specificity and 76% sensitivity for the identification of basal-like breast cancers. According to this theory, we separated 91 patients into Basal-like breast cancer group which includes 65 patients and Normal-like breast cancer group which includes 26 patients. Some studies have described mammography and ultrasound findings of TNBC, however, there haven’t been any further study to analyse the image characteristics of the TNBC subtypes. At least 90% of BBCs are invasive ductal cancers with a high mitotic index, central necrotic zones and pushing borders [32, 33]. We supposed that there must be some different features between BBCs and NBCs according to their different pathological findings.

TNBC subtypes showed differential sensitivities to cisplatin, bicalutamide (an androgen receptor antagonist used in prostate cancer), and PI3K/mTOR inhibition [2]. So differentiating BBC from NBC shows great significance in clinical work. Basal-like breast cancers are those which expressing both CK5/6 and EGFR, higher CK5/6 and EGFR expression demonstrated significantly more often central nervous system and lung recurrence but very rarely to the bones and liver [34]. BBCs always have poor prognosis, and are difficult in treatment because of lacking effective targeted therapies [17–18, 35] However, BBCs respond to chemotherapy using anthracyclines and taxanes fortunately [19]. Although BBCs response better to chemotherapy, there are more and more chemo-resistant CD44+/CD24− TNBC populations because of their innate heterogeneity. Thus leading to phenotype switching and emerging as more aggressive chemo resistant metastatic cells [36].

Mammography is the most useful way when detecting breast diseases. However, this principle was not appropriate for TNBCs. In our research, we defined 4 patients as normal after reading their mammograms. 1 BBC and 3 NBCs were undiagnosed by mammography. As we mentioned above, calcifications are barely seen in TNBCs, what’s more, imaging modalities nowadays even MRI will miss small foci of disease. Thus mammography was of limited value for TNBCs. From our study, TNBCs always found as a mass on mammography (BBCs 47/64, NBCs 16/23) and this had no significant difference between BBCs and NBCs. Most of BBCs showed breast of scattered fibroglandular (34/64) or heterogeneously dense (28/64). Almost all the NBCs showed heterogeneously dense (15/23). The borders between BBCs and NBCs had significant difference (*P* = 0.024). BBCs always showed microlobulated (30/64) or spiculated (25/64) margins. NBCs margins were almost presented as microlobulated (17/23).

It is reported that the tumour size measured by ultrasound has a relatively good correlation with the tumour size on pathology [37]. In our study TNBCs had large tumour size measured by ultrasound. Basal-like breast cancers were larger than normal-like subtype in mean size. This may be related to BBCs highly malignant character and invasive feature. Through ultrasound examination, the angular/spiculated margins always occurred in BBCs group compared with NBCs group, we analysed that for BBCs were more aggressive than NBCs and grew fast in a short period, the growth speed couldn’t be consistent with the growth of every direction. NBCs margins likely to be seen as lobulated. BBCs were more likely to be seen as markedly hypo-echoic lesions (44/65, 67.7%). Because of rapidly growing and their blood supply was insufficient and that would result in necrosis. Necrosis in BBCs was typically seen as a markedly hypo-echoic pattern on ultrasound. Also, we found that BBC was less to show posterior attenuating, which is similar to other subtypes of high-grade tumours [38]. The growth orientation of two groups had no significant differences. Malignant lesions of breast were likely to be taller than wider. However, the efficacy of ultrasound largely depends on the operator’s skill [39]. Furthermore, we also found 15 NBCs had microcalcifications whereas only one calcification case in BBCs. We supposed the reason to BBCs directly developed into an invasive stage in a short time so without a precancerous stage which could express some distinctive features. In our study, ultrasound can find lesions of the patients who appeared as normal in mammography. Ultrasound can be used to differentiate malignant and benign lesions, to guide biopsies, to assist in the selection of the appropriate therapeutic method and is of great significance to the further treatment decision. Although mammography is a gold standard for breast cancer, mammography combined with ultrasound may become useful tools to decrease the rate of missed diagnosis.

There are some limitations of our study. The one is that the number of patients is relatively small compared with other breast studies. TNBCs account for only 10–17% of all breast cancers, BBCs which are defined by gene expression microarray analysis, account for about 15% of all breast cancers [40–44]. There is a great deal of overlap between TNBCs and BBCs. So the limited number of patients in our study is unavoidable. Another is that we didn’t use magnetic resonance imaging (MRI) in our research. Dogan et al. (2010) investigated the features of TNBC by mammography, ultrasound and magnetic resonance imaging (MRI) and they found TNBC were visualised by MRI in all TNBC cases. Further study of MRI on TNBCs and the effectiveness of image prediction need to be done.

## CONCLUSIONS

Ultrasound was a more useful non-invasive tool than mammography when observing TNBCs. Larger size, angular or spiculated margins and noncalcification. These features in ultrasonography and mammography suggest BBCs, thus we can provide more suggestive information to the next treatment.
